# Odontogenic Myxoma Associated to Unerupted Mandibular Molar in a Pediatric Patient: A New Case Description with Comprehensive Literature Analysis

**DOI:** 10.3390/children12020158

**Published:** 2025-01-28

**Authors:** Marta Forte, Giuseppe D’Albis, Antonio d’Amati, Giuseppe Ingravallo, Luisa Limongelli, Gianfranco Favia, Adriano Di Grigoli, Anna Montaruli, Daniela Di Venere, Massimo Corsalini, Saverio Capodiferro

**Affiliations:** 1 Department of Interdisciplinary Medicine, University of Bari “Aldo Moro”, 70100 Bari, Italy; giuseppe.dalbis@uniba.it (G.D.); luisa.limongelli@uniba.it (L.L.); gianfranco.favia@uniba.it (G.F.); a.grigoli@hotmail.com (A.D.G.); annachiara.montaruli@gmail.com (A.M.); daniela.divenere@uniba.it (D.D.V.); saverio.capodiferro@uniba.it (S.C.); 2 Department of Precision and Regenerative Medicine and Ionian Area (DiMePRe-J), University of Bari “Aldo Moro”, 70100 Bari, Italy; antonio.damati@uniba.it (A.d.); giuseppe.ingravallo@uniba.it (G.I.)

**Keywords:** odontogenic tumors, odontogenic myxoma, mandible, retained tooth, pediatric patient

## Abstract

**Background/Objectives**: Odontogenic tumors in pediatric patients are uncommon; among all, the intraosseous occurrence of odontogenic myxoma is very rare, accounting for almost 8.5–11.6% of all odontogenic tumors in children. The radiological appearance is highly variable and is often responsible for the diagnostic delay and treatment. **Methods**: We report a case of odontogenic myxoma occurring in the posterior mandible of a 12-year-old female, found on a panoramic radiograph performed for the delayed eruption of the second inferior molar, treated by conservative surgery. A comprehensive analysis of the literature was also carried out. **Results**: The radiological features of the presented case were unique, as the lesion was encompassed within the uncompleted (developing) dental crown still unerupted, as confirmed by the macroscopic appearance. Then, the differential diagnosis included odontogenic fibroma, immature dental pulp or follicle from a developing tooth, and ameloblastic fibrodontoma. The histological examination led to the final diagnosis of odontogenic myxoma. As for the literature analysis, after a PRISMA-based selection of the papers, a total of 23 articles (case reports and case series on odontogenic myxomas in pediatric patients, a total of 33 cases) were finally selected and studied; all the pertinent data are widely discussed within the paper. **Conclusions**: The current case highlights the importance of the radiological investigation in pediatric patients when a delayed eruption lasts for several months, leading to an early diagnosis necessary to avoid more aggressive surgical therapies and possible recurrence; data from the literature about site of occurrence, sex, age, kind of surgical procedure, and recurrence rate are discussed too.

## 1. Introduction

Myxomas were first described by Virchow in 1863 as a group of rare benign neoplastic lesions characterized by local invasiveness and histologically similar to the mucinous substance of the umbilical cord [1,2]. Generally, myxomas may be found in various parts of the human body, such as the skin, subcutaneous tissue, and heart; however, their occurrence in the head and neck region is poorly documented in the literature [3].

Odontogenic myxoma (OM) of the jaw was later described in 1947 by Thoma and Goldman as benign but locally invasive tumors arising from primordial mesenchymal tooth-forming tissues [4]. They account for approximately 3.3–15.7% of all odontogenic tumors in adults and 8.5–11.6% in children. According to the World Health Organization (WHO), OMs are classified as intraosseous benign but infiltrative neoplasms derived from mesenchymal and/or odontogenic ectomesenchyme, with or without odontogenic epithelium [5].

The recurrence rate is approximately 25%, and although smaller lesions are generally asymptomatic, clinical manifestations may include facial deformity due to bone expansion without associated pain or inflammation. No metastases have been reported, and malignant transformations are rare [1]. Diagnosis is typically made in the second or third decade of life, affecting both males and females [6]. The radiological workup surely plays a crucial role in the diagnosis; OMs may variably appear on a radiograph as unilocular or multilocular radiolucency, also including trabeculae with irregular margins, with the aspect of “soap bubble”, “honeycomb”, or “tennis racquet”, and associated with tooth displacement in almost 26% of cases [1,7,8].

Histopathological features include a heterogeneous cellular population; however, several studies have demonstrated that these neoplasms are mainly composed of actively secreting fibroblastic cells, along with a significant number of myofibroblasts [7,8,9]. The whitish-grey mass is rarely encapsulated and contains spindled or stellate-shaped cells within a mucoid-rich intercellular background [9]. Immunohistochemical investigations show positivity for vimentin and SMA, while negativity for desmin, neuron-specific enolase, glial fibrillary acidic protein, and S100 may help differentiate OMs from myosarcomas in doubtful cases [9,10].

There is a lack of evidence-based consensus on the treatment modalities of OMs, mainly when occurring in young patients, as determining the need for a more radical approach to prevent recurrence remains a challenge. The surgical management varies depending on the lesion site and dimension, the patient’s age, and the clinical status. Conservative approaches include enucleation and curettage, while more radical options involve segmental mandibulectomy and mandibular reconstruction with microsurgical flaps. Additionally, the management of young patients often requires the necessity of a well-organized institution with trained surgeons and anesthetists to reduce surgical times and post-operative complications [7,8,9,10]. Despite the generally favorable prognosis, patients need close monitoring due to the high recurrence rate [8].

This study aims to report a case of OM in a 12-year-old patient, describe the diagnostic and therapeutic protocol, and discuss the diagnostic and treatment options based on a review of the literature.

## 2. Materials and Methods

This study of a 12-year-old patient includes clinical, diagnostic, and therapeutic data, along with the treatment protocol used.

A comprehensive literature search was conducted using PubMed, Scopus, and Web of Science databases to identify studies on OMs in pediatric patients. Additional manual searches were performed in journals such as Oral Diseases, Journal of Oral Pathology & Medicine, International Journal of Pediatric Dentistry, Pediatric Dentistry, and Oral Surgery, Oral Medicine, Oral Pathology and Oral Radiology (OOOO). The search utilized a combination of keywords and Medical Subject Headings (MeSH) terms: ((Odontogenic myxoma [Mesh] OR (Myxoma) OR (Myxoid tissue) OR (Fibromyxoid tissue)) AND ((Pediatric dentistry [Mesh] OR (Infant oral health) OR (Child))).

Only case reports or case series of OMs in pediatric patients reporting data on age, tumor site, localization, therapy, follow-up duration, and recurrence were included. Articles in English published between 1980 and 2024 were considered, excluding review articles, letters to editors, and animal studies. Full-text availability was a prerequisite. Two independent reviewers screened titles and abstracts to identify relevant studies, and full texts were evaluated based on inclusion criteria. Extracted data included author names, publication year, country of origin, study design, myxoma localization, patient age, surgical treatment, follow-up period, and recurrence.

### 2.1. Case Presentation

**Figure 1:**
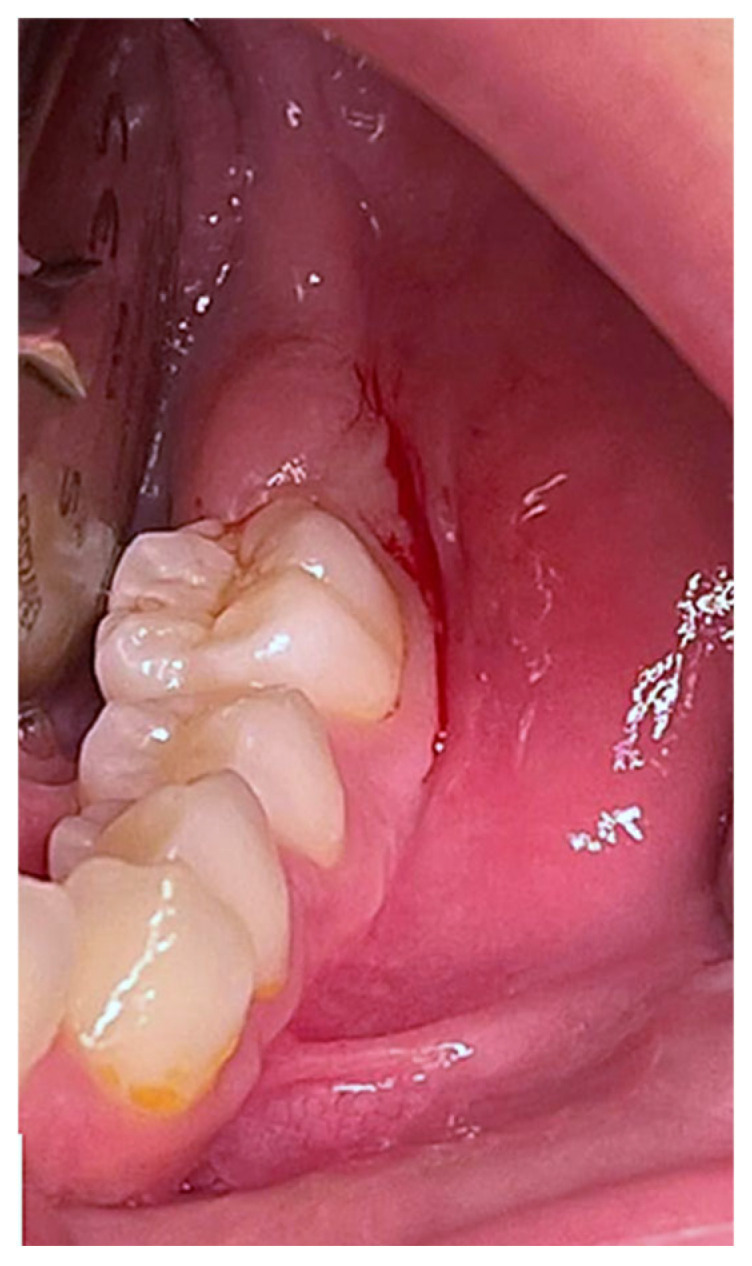
Clinical image showing a slight vestibular swelling of the left posterior mandible in the absence of tooth #3.7.

**Figure 2:**
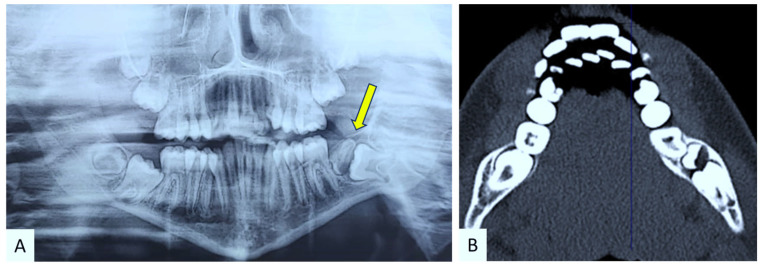
Panoramic radiograph showing a radiopaque/radiolucent lesion overlining a retained tooth (better highlighted in yellow) (**A**) consisting of the developing tooth crown with a radiopaque appearance encompassing the radiolucent lesion, better defined on CT scan (**B**).

In January 2024, a 12-year-old female patient presented to the Complex Unit of Odontostomatology at the University of Bari “Aldo Moro”—Italy, with a slight intraoral painless swelling in the vestibular area of the left posterior mandible, associated with missing tooth in #37 position (Figure 1). At the panoramic radiogram, the lesion showed a radiologically mixed (radiopaque-radiolucent) appearance above a retained tooth, better defined on CT scans showing a developing tooth crown (the radiopaque counterpart) encompassing a discoid lesion (the radiolucent counterpart) (Figure 2). The patient’s medical history was unremarkable.

**Figure 3:**
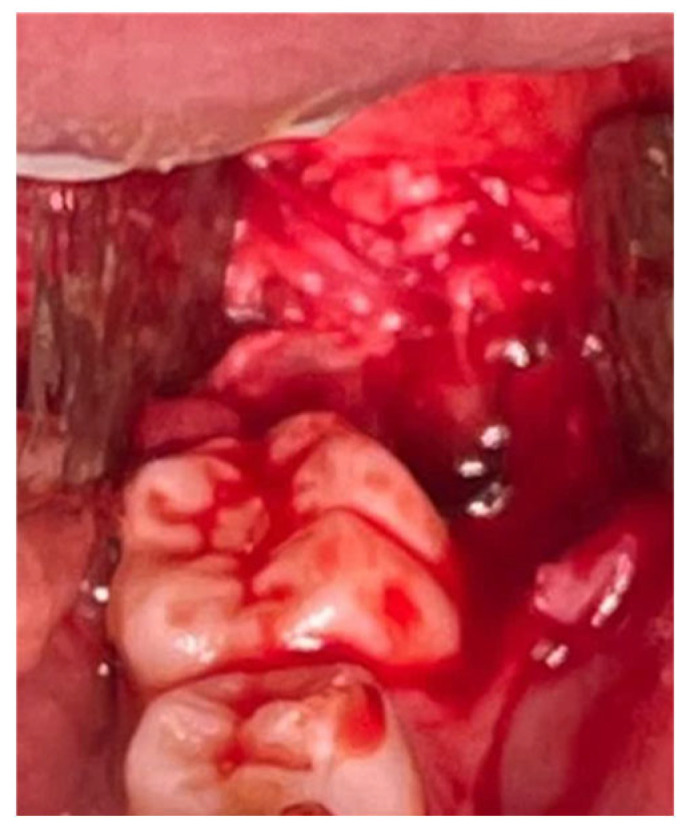
Surgical elevation of the mucoperiosteal flap and lesion excision along with the incomplete crown with preservation of the unerupted tooth.

**Figure 4:**
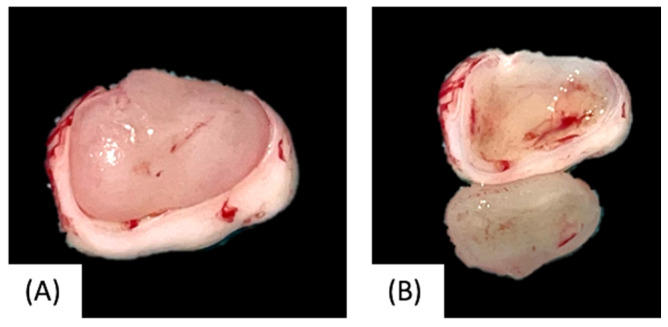
Macroscopic appearance of the surgical specimen within the uncompleted tooth crow (**A**) and the tooth crow alone (**B**).

**Figure 5:**
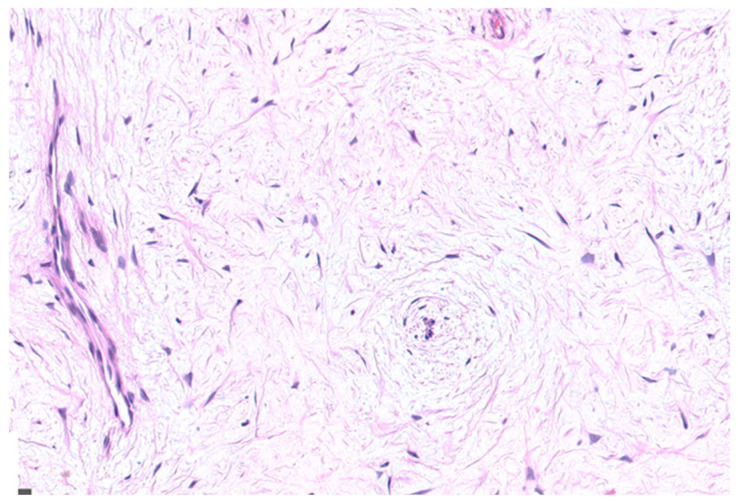
Microscopic examination showing stellate and spindle-shaped cells haphazardly arranged in a loose myxoid stroma. Rare small islands of inactive odontogenic epithelial rests were also present (HE, 200×).

Based on the clinical and radiological features, surgical excision with enucleation and curettage was performed under general anesthesia, with preservation of the retained tooth (Figure 3). Postoperative pain control included paracetamol (500 mg twice daily for 3–4 days). The surgical specimen, consisting of a white-greyish mass of hard consistency, was immediately fixed in 10% formalin and sent for histopathological examination (Figure 4). Histological findings showed stellate and spindle-shaped cells within a loose myxoid stroma, along with rare small islands of inactive odontogenic epithelial rests (Figure 5), leading to the final diagnosis of OM.

**Figure 6:**
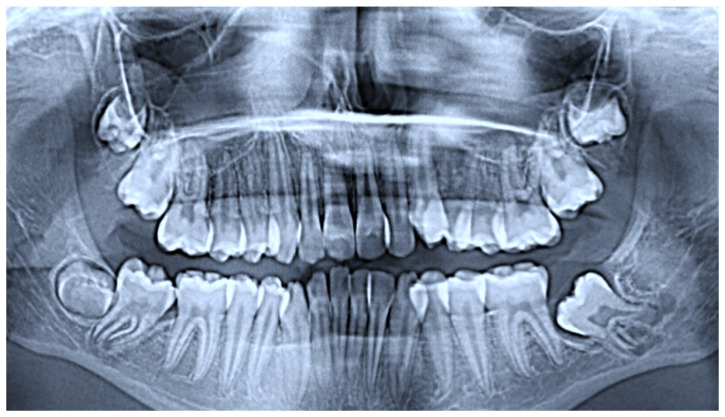
Panoramic radiograph at a 10-month follow-up.

No postoperative complications were observed, soft tissues healed completely in two weeks, and no recurrence was observable at a 10-month radiological follow-up (Figure 6). The patient continues periodic monitoring also for orthodontic purposes.

### 2.2. Literature Analysis

**Figure 7:**
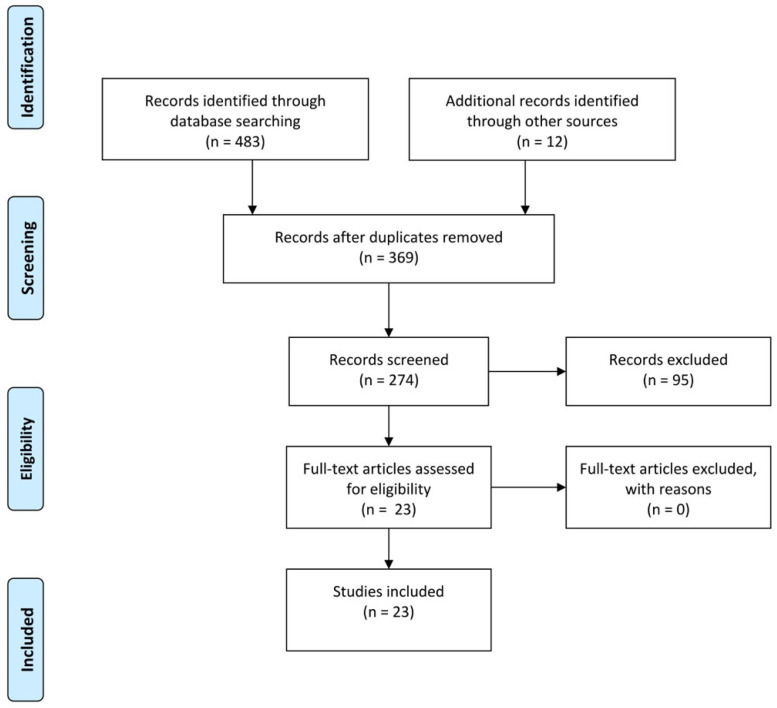
Flow diagram of the study selection process.

The literature research identified 483 articles, including 58 reviews, three systematic reviews, 259 case reports, and 15 case series. A detailed summary of the articles’ screening process and eligibility criteria is presented in Figure 7.

After an initial screening of 369 article titles, 274 abstracts were selected for further review. The study focused solely on case reports and case series of OMs in pediatric patients. Only articles published in English between 1980 and 2024 were considered, excluding review articles, letters to editors, and animal studies. After the screening, 23 articles met the inclusion criteria and were analyzed.

## 3. Results

Extracted data focused on patient age, tumor location, treatment, follow-up duration, and recurrence are summarized in Table 1.

## 4. Discussion

Herein, we report a rare occurrence of OM in the mandible of a pediatric patient, specifically focusing on its management from diagnosis to follow-up, as well as a comparative analysis of our case with data from the pertinent literature.

### 4.1. Epidemiology

OMs represent approximately 3.3–15.7% of all odontogenic tumors in adults and 8.5–11.6% in the pediatric population, with a recurrence rate of about 25%. They generally occur in both males and females, most commonly in the second or third decade of life. In accordance with the literature, our case describes an occurrence in a 12-year-old female patient.

### 4.2. Clinical-Radiological Features and Differential Diagnosis

Among the most described clinical manifestations of OMs, pain or inflammation is usually absent. Only in advanced cases might they include facial deformity due to the expansion of the maxillary bone. Metastases have never been reported, and malignant transformations are exceedingly rare. Radiological workup plays a crucial role in diagnosis; OMs may variably appear on radiographs as unilocular or multilocular radiolucencies, often featuring trabeculae with irregular margins, with typical appearances such as “soap bubble”, “honeycomb”, or “tennis racquet”. In approximately 26% of cases, these lesions are associated with tooth displacement [1,7,8]. On macroscopic examination, OMs appear as whitish-grey masses that are rarely encapsulated. Microscopically, they are characterized by spindled or stellate-shaped cells (mainly actively secreting fibroblastic cells, along with a significant number of myofibroblasts) within a mucoid-rich intercellular background [9]. The radiological features of the presented case were unique, as the lesion was encompassed within the incomplete (developing) dental crown, which remained unerupted, as confirmed by macroscopic examination. Differential diagnoses included odontogenic fibroma, immature dental pulp or follicle from a developing tooth, and ameloblastic fibrodontoma. Histological examination ultimately confirmed the diagnosis of OM.

### 4.3. Odontogenic Myxoma in Pediatric Patients: Analysis of the Relevant Literature

A total of 23 articles were included in this analysis, consisting of case reports and case series on odontogenic myxomas in pediatric patients. These studies focused on factors such as patient age, tumor site and localization, treatment approach, follow-up duration, and recurrence. In the maxilla, 21 cases were reported with the majority localized to the paranasal region. For instance, Kiresur MA et al. reported a 17-year-old patient with a posterior maxillary tumor treated by resection, with a 6-month follow-up and no recurrence [11]. Similarly, Wankhedkar et al. described an 8-year-old patient with an anterior maxillary tumor treated by biopsy and subsequent partial maxillectomy, with no recurrence after 6 months [1]. Toro et al. documented a rare case of a 29-month-old patient with a tumor in the maxillary sinus, treated with biopsy and curettage. Follow-up at 2 months showed no recurrence [6]. A case series by Kansy et al. described two patients, aged 12 months and 11 months, respectively, both with paranasal maxillary tumors; the first patient underwent enucleation and curettage with a 2-year follow-up and no recurrence, while the second required partial maxillectomy and experienced recurrence within 0.16 years [10]. Fenton et al. reported a 17-month-old patient with a maxillary tumor treated via lateral rhinotomy, with no recurrence observed over a 16-month follow-up [12]. King et al. presented two cases of 17- and 18-month-old patients, both treated with enucleation. Neither patient showed recurrence after follow-up periods of 18 and 24 months [13]. Rotenberg et al. reported a case series of three patients with maxillary tumors treated through different surgical approaches, including lateral rhinotomy and medial maxillectomy. Follow-up periods ranged from 4 to 14 years, with no recurrences noted in any case [14]. Wachter et al. also presented two cases treated by excisional biopsy; patients aged 13 and 19 months were followed for 24 months with no evidence of recurrence [15]. Sasidhar Singaraju et al. reported a 7-year-old patient with a right maxillary tumor extending into the sinus, treated by resection, with no recurrence after 6 months [16]. Similarly, Karuna Jindwani et al. and Vjieev Vasudevan et al. reported cases of 10- and 13-year-old patients with posterior maxillary tumors treated with resection, though no follow-up data were provided [17,18]. Harokopaki Hajishengallis et al. documented a 6-year-old patient with a posterior maxillary tumor treated by resection, with no recurrence after 9 months [8]. Kadlub et al. reported four cases of paranasal maxillary tumors in children aged 14 to 23 months. Three cases were treated by enucleation and curettage, with two experiencing recurrences within 1 to 1.5 years [19]. Brewis et al. described a 13-month-old patient with a paranasal maxillary tumor treated by enucleation and curettage, with no recurrence after 0.3 years [20]. Iatrou et al. presented a similar case of a 12-month-old treated by the same method, with no recurrence after 3.5 years [21]. James and Lucas and Leiberman et al. reported cases of paranasal tumors treated by enucleation or resection, all showing no recurrence during follow-up periods ranging from 0.5 to 0.7 years [22,23].

With regard to the occurrence in the mandible, Mauro et al. described a 6-year-old patient treated with conservative surgery with no recurrence after 6 months [26]. Landes et al. reported a 14-year-old patient treated with radical surgery, showing no recurrence after 30 months [27]. Li et al. presented a case series of two patients aged 7 and 12: one was treated conservatively, and the other with radical surgery. Follow-up periods of 84 and 36 months showed no recurrences [28]. Finally, Lo Muzio et al. described a 16-year-old patient with a mandibular tumor treated with conservative surgery, with no recurrence after a 31-month follow-up [29].

The distribution of tumor site (maxilla or mandible) and localization (posterior, anterior, paranasal, etc.) was examined to provide a clear understanding of the myxomas characteristics in pediatric patients:

- Maxilla: Of the 33 cases, 29 cases (88%) were localized in the maxilla.
- Mandible: Only 4 cases (12%) involved the mandible.

This distribution shows a significantly higher incidence of OMs in the maxilla compared to the mandible.

### 4.4. Myxomas Localization Within the Maxilla

- Paranasal Region: Among the maxillary cases, the paranasal region was the most common site, with 15 cases (52%) involving this area.
- Posterior Maxilla: 5 cases (17%) were found in the posterior maxilla.
- Anterior Maxilla: Only 1 case (3%) was localized in the anterior maxilla.
- Maxillary Sinus: 2 cases (7%) were specifically located in the maxillary sinus.
- Not Specified (NS): In 6 cases (21%), the specific localization within the maxilla was not clearly specified.

### 4.5. Myxomas Localization Within the Mandible

Of the 4 cases in the mandible:

- 2 cases (50%) did not have a specified localization within the mandible.
- 2 cases (50%) were located in unspecified regions, though described as either posterior or not clearly localized.

The breakdown reveals that the maxilla is overwhelmingly the most common site for OMs in pediatric patients, accounting for 88% of the cases. Within the maxilla, the paranasal region is the most frequently affected area, representing over half of the cases (52%). The posterior maxilla and maxillary sinus are less commonly affected, while the anterior maxilla is the least common site, occurring in only 3% of cases.

In contrast, the mandible is much less frequently involved, with only 12% of the total cases localized there and with limited specification regarding precise myxoma localization in these cases.

In the occurrence of OMs in children, this analysis underscores the predominance of the maxilla, particularly the paranasal region.

An analysis was also conducted to assess the correlation between treatment strategies and recurrence rates in pediatric patients with odontogenic myxomas.

### 4.6. Treatment Approaches

The main treatment modalities included:

- Resection: Used in 8 cases (24%).
- Enucleation and Curettage: Applied in 12 cases (36%).
- Conservative Surgery: Reported in 3 cases (9%).
- Biopsy or Partial Maxillectomy: Used in 2 cases (6%).
- Radical Surgery: Applied in 3 cases (9%).
- Lateral Rhinotomy/Medial Maxillectomy: Applied in 5 cases (15%).

### 4.7. Recurrence Rates

Out of the 33 cases:

- Recurrence occurred in 4 cases (12%), while 29 cases (88%) had no recurrence during the follow-up period.
- The recurrence cases were linked to the following treatments:
- Partial Maxillectomy (1 case): Recurrence occurred within 0.16 years.
- Enucleation and Curettage (3 cases): Recurrences occurred in patients with follow-up periods of 1 to 1.5 years.

### 4.8. Treatment and Recurrence Correlation

- Resection: None of the 8 cases treated with resection showed recurrence, suggesting this approach may be more effective in preventing tumor regrowth.
- Enucleation and Curettage: Despite being used in 12 cases, 3 of these (25%) experienced recurrence, indicating a higher likelihood of tumor regrowth compared to other methods.
- Conservative Surgery: No recurrences were observed in the 3 cases treated with conservative surgery, though this was used less frequently.
- Radical Surgery: All 3 cases treated with radical surgery showed no recurrence, indicating a potential advantage of more aggressive surgical approaches.

The analysis indicates that resection and radical surgery had the lowest recurrence rates, with none of the cases showing tumor regrowth. In contrast, enucleation and curettage were associated with a higher recurrence risk, observed in 25% of cases. Conservative surgical approaches also appeared effective, though the sample size for this treatment was smaller. Therefore, more aggressive surgical approaches, such as resection and radical surgery, may reduce the likelihood of recurrence in pediatric OMs.

Overall, most cases involved maxillary tumors, most commonly in the paranasal region, and were treated with surgery such as resection, enucleation, or curettage. Recurrences were rare, with only a few cases reporting tumor regrowth within a short follow-up period. Longer follow-ups generally showed successful outcomes without recurrence.

### 4.9. Therapeutic Management in Paediatric Patients

The literature lacks evidence-based consensus regarding the treatment protocol of OMs, especially when pediatric patients are involved. Surgical management is still challenging for clinicians as conservative approaches are safer for patients, although associated with a significantly higher recurrence rate than radical interventions. Generally, a conservative approach consists of enucleation and curettage, whereas advanced cases need a more radical approach also till to the segmental mandibulectomy and reconstruction. Finally, as the recurrence rate is reported to be extremely high, a very close follow-up is recommended, although the overall prognosis is good. In some cases, authors described conservative excision of margins or planes as the gold standard, whereas others recommend more radical resection in order to minimize the risk of recurrence.

This current case emphasizes the value of radiological evaluation of whether a retained tooth persists for several months since this may be associated with pathological implications that require an early differential diagnosis to prevent more drastic surgical treatments and potential recurrence, particularly in young patients. Collected data summarized in this study are not sufficient to assess a surgical protocol for the treatment of such odontogenic lesions in pediatric patients as in the choice of the best surgical approach, many factors must be considered, such as localization of the tumor, as it determines the excision’s extension, age of the patient and anesthesiologic regimen, association, or not with teeth and radiographically visible margins [8].

## 5. Conclusions

This case and extracted data from the comprehensive literature analysis underscore the necessity of tailored surgical approaches based on clinical and radiological findings to minimize recurrence and complications in pediatric OMs.

## Figures and Tables

**Table 1:** Data of pertinent literature of odontogenic myxomas in paediatric patients.

First Author (Year)	Study Design—No. Cases	Site	Localization	Age	Treatment	Follow Up	Recurrence
**Kansy et al. (2012) [10]**	Case series—2	Maxilla	Paranasal	12-months-old	Enucleation + curettage	2 y	No
		Maxilla	Paranasal	11-months-old	Partial maxillectomy	0.16 y	Yes
**Kiresur MA et al. (2014) [11]**	Case report—1	Maxilla	Posterior	17-years-old	Resection	6 m	No
**Wankhedkar et al. (2018) [1]**	Case report—1	Maxilla	Anterior maxilla	8-years-old	Biopsy + partial maxillectomy	6 m	No
**Toro et al. (2015) [6]**	Case report—1	Maxilla	Maxillary sinus	29-months-old	Biopsy + curettage	2 m	No
**Fenton et al. (2003) [12]**	Case report—1	Maxilla	NS	17-months-old	Lateral rhinotomy	16 m	No
**King et al. (2008) [13]**	Case series—2	Maxilla	NS	18-months-old	Enucleation	18 m	No
		Maxilla	NS	17-months-old	Enucleation	24 m	No
**Rotenberg et al. (2004) [14]**	Case series—3	Maxilla	NS	13-months-old	Lateral rhinotomy	4 y	No
		Maxilla	NS	18-months-old	Medial Maxillectomy	14 y	No
		Maxilla	NS	16-months-old	Lateral rhinotomy	7 y	No
**Wachter et al. (2003) [15]**	Case series—2	Maxilla	NS	13-months-old	Excisional biopsy	24 m	No
		Maxilla	NS	19-months-old	Excisional biopsy	24 m	No
**Sasidhar Singaraju et al.** **(2010) [16]**	Case report—1	Maxilla	Right maxilla+sinus	7-years-old	Resection	6 m	No
**Karuna Jindwani et al.** **(2015) [17]**	Case report—1	Maxilla	Posterior	10-years-old	Resection	NS	NS
**Vjieev Vasudevan et al.** **(2011) [18]**	Case report—1	Maxilla	Posterior	13-years-old	Resection	NS	NS
**Harokopaki Hajishengallis et al. (2006) [8]**	Case report—1	Maxilla	Posterior	6-years-old	Resection	9 m	No
**Kadlub et al. (2014) [19]**	Case series—4	Maxilla	Paranasal	18-months-old	Enucleation + curettage	6 y	No
		Maxilla	Paranasal	23-months-old	Enucleation + curettage	4 y	No
		Maxilla	Paranasal	21-months-old	Enucleation + curettage	1.5 y	Yes
		Maxilla	Paranasal	14-months-old	Enucleation + curettage	1y	Yes
**Brewis et al. (2000) [20]**	Case report—1	Maxilla	Paranasal	13-month-old	Enucleation + curettage	0.3 y	No
**Iatrou et al. (2010) [21]**	Case report—1	Maxilla	Paranasal	12-months-old	Enucleation + curettage	3.5 y	No
**James and Lucas. (1987) [22]**	Case series—2	Maxilla	Paranasal	11-month-old	Enucleation + curettage	0.7 y	No
**Leiberman et al. (1990) [23]**	Case series—2	Maxilla	Paranasal	18-months-old	Resection	0.5 y	No
		Maxilla	Paranasal	15-months-old	Resection	NS	NS
**Prasannan et al. (2005) [24]**	Case report—1	Maxilla	Paranasal	20-months-old	Enucleation	0.5 y	No
**Rios Y Valles-Valles et al. (2012) [25]**	Case report—1	Maxilla	Paranasal	11-months-old	Enucleation + curettage	4 y	No
**Mauro et al. (2012) [26]**	Case report—1	Mandible	NS	6-years-old	Conservative surgery	6 m	No
**Landes et al. (2008) [27]**	Case report—1	Mandible	NS	14-years-old	Radical surgery	30 m	No
**Li et al. (2006) [28]**	Case series—2	Mandible	NS	12-years-old	Radical surgery	36 m	No
		Mandible	NS	7-years-old	Conservative surgery	84 m	No
**Lo Muzio et al. (1996) [29]**	Case report—1	Mandible	NS	16-years-old	Conservative surgery	31 m	No

NS: not specified.

## Data Availability

The data collected in the current study were downloaded from the following databases: PubMed (https://pubmed.ncbi.nlm.nih.gov), Scopus (https://www.scopus.com), and Web of Science (https://clarivate.com/academia-government/scientific-and-academic-research/research-discovery-and-referencing/web-of-science/).
